# Artificial Intelligence Applications in Diagnosing and Managing Non-syndromic Craniosynostosis: A Comprehensive Review

**DOI:** 10.7759/cureus.45318

**Published:** 2023-09-15

**Authors:** Amna Qamar, Shifa F Bangi, Rajas Barve

**Affiliations:** 1 Surgery, John Radcliffe Hospital, Oxford, GBR; 2 Medicine and Surgery, University Hospitals of Leicester, Leicester, GBR; 3 General Surgery, Bedford Hospital, Bedford, GBR

**Keywords:** computer-aided design and computer-aided manufacturing (cad/cam), deep learning algorithms, machine learning algorithms, artificial intelligence, craniosynostosis, pediatric craniofacial surgery

## Abstract

Craniosynostosis is characterised by the premature fusion of one or more cranial sutures, resulting in an abnormal head shape. The management of craniosynostosis requires early diagnosis, surgical intervention, and long-term monitoring. With the advancements in artificial intelligence (AI) technologies, there is great potential for AI to assist in various aspects of managing craniosynostosis.

The main aim of this article is to review available literature describing the current uses of AI in craniosynostosis. The main applications highlighted include diagnosis, surgical planning, and outcome prediction.

Many studies have demonstrated the accuracy of AI in differentiating subtypes of craniosynostosis using machine learning (ML) algorithms to classify craniosynostosis based on simple photographs. This demonstrates its potential to be used as a screening tool and may allow patients to monitor disease progression reducing the need for CT scanning. ML algorithms can also analyse CT scans to aid in the accurate and efficient diagnosis of craniosynostosis, particularly when training junior surgeons. However, the lack of sufficient data currently limits this clinical application.

Virtual surgical planning for cranial vault remodelling using prefabricated cutting guides has been shown to allow more precise reconstruction by minimising the subjectivity of the clinicians’ assessment. This was particularly beneficial in reducing operating length and preventing the need for blood transfusions.

Despite the potential benefits, there are numerous challenges associated with implementing AI in craniosynostosis. The integration of AI in craniosynostosis holds significant promise for improving the management of craniosynostosis. Further collaboration between clinicians, researchers, and AI experts is necessary to harness its full potential.

## Introduction and background

Craniosynostosis is the premature fusion of cranial sutures altering bone growth and resulting in an abnormal head shape [1]. Epidemiological studies show the incidence to be approximately 1 in 1,700 live births [1]. Craniosynostosis can be classified as syndromic or non-syndromic, with the latter encompassing the majority of cases [2]. Although the aetiology of craniosynostosis is yet to be fully elucidated, several hypotheses exist. Some studies have linked maternal factors such as a bicornuate uterus, causing inter-uterine constraint, or sodium valproate use during pregnancy with increased risk of craniosynostosis [2]. More recently, several gene mutations have been identified in association with synostosis. These include the *fibroblastic growth factor receptor* (*FGFR*) and *TWIST *genes, largely inherited in an autosomal dominant pattern; however, these have been seen sporadically in non-syndromic craniosynostosis [2]. Altered bone growth at the fused sutures and compensatory growth at the unaffected sutures result in an abnormal head shape [3]. Each suture involvement results in a distinct head shape so craniosynostosis is a clinical diagnosis. However, CT scanning with 3D reconstruction is the gold standard as it allows visualisation of any fused sutures and any obvious cerebral structural abnormalities [2]. These can then be further assessed through MRI. Genetic testing may be offered for the family to better plan any subsequent children [4].

There are numerous surgical approaches for correcting head shape. Although the main aim is to correct appearance, preventing complications forms part of the indication [3]. These include increased intracranial pressure, visual impairment, hearing loss, and developmental delay, among others [2]. The timing of surgery is typically within the first six to 12 months of life [3]. There are predominantly two types of operative options, namely, endoscopic and open vault remodelling [3]. Postoperatively, dynamic skull growth continues; this is accounted for and relied upon to achieve a good aesthetic outcome. However, this growth can be unpredictable and may lead to a worsened appearance [5]. Therefore, a proportion of patients will require subsequent surgery for further corrections [5]. As such, some units are exploring the use of artificial intelligence (AI) in the diagnosis and management of craniosynostosis.

Modern machine learning (ML) algorithms have been able to achieve the diagnosis of synostosis using 2D photographs and 3D stereophotogrammetry eliminating the variability of human pattern recognition [6]. ML is a branch of AI in which the algorithms are trained to perform a function without being explicitly coded for. ML algorithms become more accurate and consistent when provided with more datasets [7]. Deep learning algorithms are a subset of ML and can automatically extract features from the input data to perform the function [7].

## Review

Methodology

This literature review was conducted using the PubMed database using search terms “craniosynostosis” and “artificial intelligence” and “virtual”. Only articles about non-syndromic craniosynostosis were included. A decision was made to make this study a narrative review rather than a systematic review or meta-analysis due to the paucity of literature on this topic.

Use of artificial intelligence in the diagnosis of craniosynostosis

**Figure 1:**
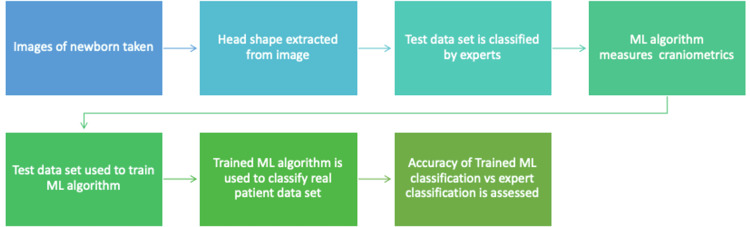
A flow diagram illustrating the method of processing images, training algorithms, and testing these algorithms to accurately diagnose craniosynostosis. ML: machine learning

Several studies have used a similar methodology of collecting photographs or CT scans that are processed using programmes such as GrabCut and MATLAB to isolate the head shape from the images [7-13]. A test dataset, classified by expert physicians, is used to train the ML algorithm to measure cranial indices which are then used to classify the type of synostosis [7-13]. The accuracy of the algorithm is then assessed by running it on a separate dataset [7-13]. This is demonstrated in Table 1 and Figure 1.

**Table 1:** A summary of the studies exploring the use of machine learning and deep learning models in the diagnosis and classification of craniosynostosis.

Study	Number of patients	Imaging	Algorithm	Number of craniometrics	Classifications
Pastor et al., 2020 [8]	80 (174 images)	2D photographs	Multiple linear regression, support vector regression	2	Brachycephaly, plagiocephaly
Bookland et al., 2021 [11]	339	2D photographs	Linear discrimination model	5	Brachycephaly, lagiocephaly, sagittal, metopic, right unicoronal, left unicoronal
Sabeti et al., 2022 [12]	145	2D photographs	K-nearest neighbour, support vector machine, random forest	9	Sagittal, metopic, unicoronal
Paro et al., 2022 [7]	174	2D photographs	Random forest, classification and regression tree, linear discriminant analysis	5	Non-craniosynostosis (brachycephaly, plagiocephaly), craniosynostosis (sagittal, metopic, unicoronal)
Anderson et al., 2023 [9]	1,001	2D photographs	Classification and regression tree	2	Sagittal, other
Jong et al., 2020 [10]	196	3D stereophotogrammetry	Deep learning	n/a	Scaphocephaly, trigonocephaly, anterior plagiocephaly
You et al., 2020 [13]	50	CT	Deep learning	n/a	Sagittal (anterior, central, posterior, complex)

A study by Pastor et al. used MATLAB to process photographs of the patient and measure the cranial ratio and cranial vault asymmetry index to quantify the severity of brachycephaly and plagiocephaly, respectively [8]. They correlated this with a reference dataset from a 3D cranial scanner [8]. When using their ML model of support vector regression, they were able to identify brachycephaly and plagiocephaly with 86.7% accuracy [8]. Likewise, Anderson et al. demonstrated the use of 2D photographs to measure the posterior arc angle and the cephalic index [9]. These have been found to accurately classify sagittal craniosynostosis and have the potential to be incorporated into ML models [9]. Jong et al. also showed similar results using a deep learning algorithm and 3D photogrammetry to sub-classify patients with 99.5% accuracy [10]. These results are supported by Bookland et al. who used linear discriminant modelling to classify six types of craniosynostosis with an overall 93.3% accuracy [11]. Sabeti et al. used an ML programme to measure different cranial indices as well as statistical pattern-matching indices [12]. The most accurate (85-92%) classification was achieved by combining both types of measures [12]. Paro et al. took this further and evaluated three different ML models, namely, classification and regression tree, linear discriminant analysis, and random forest partition tree, with the latter having the best accuracy (94.8%) compared to the provider’s initial diagnosis [7].

You et al. developed a deep learning algorithm to classify subtypes of sagittal craniosynostosis using Hounsfield units to segment and analyse CT images [13]. They achieved over 90% accuracy on the test data but struggled to consistently and accurately classify subtypes once given real, unseen data [13]. They concluded that sample size and the seniority of the surgeon making the initial diagnosis are key limiting factors [13]. Similarly, Junn et al. also used an ML algorithm to classify the severity of metopic synostosis using preoperative CT scans and demonstrated its comparability to previously established craniometric measures [14].

Most of the cranial metrics used are ratio-based or angle-based measurements meaning that while the observer’s distance to the subject in the photograph does not have a big impact, the image orientation does [11]. Bookland et al. found that variation of more than 5 degrees off the midline leads to variability in the ratio-based measurement [11]. Junn et al. used specialised software to orientate the CT images of each skull in a consistent plane [14]. It is evident that finding a standardised way to ensure uniform orientation of the skull is imperative, especially for the training dataset.

Use of artificial intelligence in the treatment of craniosynostosis

In an effort to optimise surgical outcomes including preventing further corrective surgery, recent technological advancements including virtual surgical planning, computer-aided design (CAD), and computer-aided manufacturing (CAM) are being more frequently utilised. Mardini et al. used virtual surgery using CAD/CAM to create a prefabricated template to plan the osteotomies and placing of bone fragments, resulting in a more precise and rapid reconstruction, as well as a better alignment of expectations for family members [6]. In the cases of distraction osteogenesis, similar models have been able to establish surgical vectors and landmarks [15]. A study by Bozkurt et al. used a finite element model (FEM) to simulate spring-assisted cranial expansion with osteotomies [16]. An FEM, in this context, is a computational tool that divides the problem into interconnected elements, builds simulations, and allows analysis of the spring’s impact on different placements [16]. The results showed only a ±5 mm difference in the skull geometry between the models and postoperative scans [16]. It demonstrates the potential uses of FEM to simulate surgical outcomes and allow specific surgical planning [16]. These studies concur that CAM cutting guides display obvious advantages [16]. There is less inter-surgeon variability, increased reproducibility, shorter operative time and reduction in the rate of complications, primarily the need for blood transfusions [5,15,17,18].

Along with its importance in immediate surgical planning, Cross et al. demonstrated the use of FEM to compare the long-term outcomes on brain growth of three different reconstruction models [19]. Varying pressure outcomes across the brain demonstrated the potential impact of different surgical interventions on growth [19]. As AI becomes more integrated into medical practice, the use of such modelling techniques will be increasingly accessible for clinicians to use.

Discussion

It is clear that the applications of AI have rapidly progressed in recent times. Diagnosis of craniosynostosis is a sub-specialist area, making it a difficult diagnosis for most clinicians, including experienced craniofacial surgeons. Machine and deep learning models have the potential to eliminate the inter-observer variability as even the most senior surgeons can have disagreements when diagnosing the subtype of craniosynostosis. Currently, diagnosis is limited to tertiary or quaternary centres and accessibility to such clinics can lead to delays in diagnosis. ML-based models have immense potential to be used as a screening tool and for point-of-care diagnosis, streamlining the cases seen in specialist clinics and ultimately reducing their patient burden and timescale to diagnosis for patients and their families. This is particularly well illustrated by Paro et al., who used photographs taken by parents/carers and ML algorithm analysis to accurately recognise pathological head shapes [7]. Primary care providers could use this technology to identify patients who require further assessment in specialist clinics, which could be conducted via virtual or face-to-face consultations. This could be easily integrated into craniofacial services in a post-pandemic world.

Al algorithms can also be used in surgical education to train junior surgeons. Surgical correction of synostosis has various approaches; however, in all techniques, the location and size of osteotomies are critical to the final outcome [16]. Bozkurt et al. demonstrated the use of FEMs, a type of simulation modelling, to plan osteotomies and predict the surgical outcome, taking into account postoperative skull growth [16]. This technique allows for extensive surgical planning due to better visualisation of the planned osteotomies and the specific landmarks and dimensions are decided preoperatively. This will be particularly useful for junior surgeons who are inexperienced and limits the need for subjective intraoperative cranial assessment and decision-making. These techniques could be incorporated with CAD/CAM modelling to expand their application to all approaches of vault remodelling. A study by Julie et al. demonstrated that the use of CAD/CAM led to significant improvement in the surgical outcomes of younger surgeons [18]. Further investigation into the impact of utilising a combination of models will be interesting, particularly in the hands of trainee surgeons.

Nevertheless, there are clear limitations impeding the clinical implementation of AI models. One of the largest barriers is the cost and training required to launch this practice in craniofacial units. Furthermore, the development of a reliable algorithm consists of two major elements. First, the training data must be reliable as this will be the foundation for ML. The impact of accurate input data was clearly illustrated by You et al., who found models with input data from senior surgeons to be more accurate than those from junior surgeons [13]. Second, a large set of training data verified by multiple surgeons is needed to ensure reliability which is a very time-consuming endeavour [7-14]. Several different craniometrics to classify craniosynostosis have been proposed in these studies. Thus, despite similar methodologies, each study varies in its choice of image modalities, algorithms, and cranial indices. Due to this heterogeneity, there is difficulty in generating high-quality comparative studies. Guidelines with clearer definitions of each subtype using objective and reproducible craniometrics would aid the progression of this technology [1]. It would also allow the amalgamation of datasets across multiple centres, greatly improving the accuracy of the algorithms. ML algorithms are also susceptible to statistical errors such as overfitting. An example is the risk of under-sampling milder cases, as CT imaging is usually done in more severe presentations [20]. It should also be noted that certain studies reported lower accuracy when processing unseen patient data compared to test data limiting their application in the current clinical setting [13].

Given that the majority of studies in this review used 2D photographs in diagnosing craniosynostosis, as shown in Table 1, there is a clear preference for this imaging modality likely due to its accessibility and lack of radiation exposure. However, it should be noted that Bookland et al. discussed head orientation as a limitation in the widespread use of 2D photographs in diagnosis [11]. To overcome this, an app with auto face detection software such as Face ID can be developed to identify optimal head orientation allowing parents/carers to take good quality photographs of their child’s head. It may also be worth evaluating the use of X-rays as an alternative imaging modality, as described by Mizutani et al. [21]. However, their algorithm is yet to be tested on a dataset that was not used in training the algorithm itself [21].

The need for CT scanning also limits the role of CAD/CAM to more complex patients, thus very few studies have discussed its role in non-syndromic cases [15]. On the other hand, most units will have pre and postoperative medical photography for all patients for monitoring and assessing surgical outcomes. As discussed, ML algorithms can reliably diagnose synostosis using photographs [14]. García-Mato et al. reported using a portable scanning device to obtain 3D photographs postoperatively to measure surgical outcomes [5]. Increasing the accuracy of 3D modelling to the point that it is comparable to CT scan-based classification will reduce the need for CT scanning and decrease radiation exposure for the patient. Further work is needed to directly compare the accuracy of ML models that use photographs versus those that use CT scans.

## Conclusions

AI has been shown to be a useful tool in improving outcomes for patients with craniosynostosis through its modelling techniques used for diagnosis and operative planning. Due to the limited accessibility of AI, there are obvious drawbacks, including small, potentially skewed datasets, leading to algorithmic bias. There is insufficient literature to explore whether these modelling techniques are reliable in minority populations. Furthermore, the lack of standardisation in cranial indices impedes the capability to pool together global datasets to overcome these limitations. The rapid evolution of these technologies creates immense potential for the widespread clinical implementation of AI, including augmenting diagnosis, guiding operative techniques, and predicting long-term outcomes for patients.
